# Single-Cell Analysis of Antigen-Specific CD8+ T-Cell Transcripts Reveals Profiles Specific to mRNA or Adjuvanted Protein Vaccines

**DOI:** 10.3389/fimmu.2021.757151

**Published:** 2021-10-29

**Authors:** Trine Sundebo Meldgaard, Fabiola Blengio, Denise Maffione, Chiara Sammicheli, Simona Tavarini, Sandra Nuti, Roland Kratzer, Duccio Medini, Emilio Siena, Sylvie Bertholet

**Affiliations:** ^1^ Research & Development, GSK, Siena, Italy; ^2^ Biochemistry & Molecular Biology, University of Siena, Siena, Italy; ^3^ Chemical & Biological Sciences, University of Torino, Torino, Italy; ^4^ Research & Development, GSK, Rockville, MD, United States

**Keywords:** CD8+ T cells, single-cell, heterogeneity, gene expression, self-amplifying mRNA, vaccines, high-throughput

## Abstract

CD8+ T cells play a key role in mediating protective immunity after immune challenges such as infection or vaccination. Several subsets of differentiated CD8+ T cells have been identified, however, a deeper understanding of the molecular mechanism that underlies T-cell differentiation is lacking. Conventional approaches to the study of immune responses are typically limited to the analysis of bulk groups of cells that mask the cells’ heterogeneity (RNA-seq, microarray) and to the assessment of a relatively limited number of biomarkers that can be evaluated simultaneously at the population level (flow and mass cytometry). Single-cell analysis, on the other hand, represents a possible alternative that enables a deeper characterization of the underlying cellular heterogeneity. In this study, a murine model was used to characterize immunodominant hemagglutinin (HA_533-541_)-specific CD8+ T-cell responses to nucleic- and protein-based influenza vaccine candidates, using single-cell sorting followed by transcriptomic analysis. Investigation of single-cell gene expression profiles enabled the discovery of unique subsets of CD8+ T cells that co-expressed cytotoxic genes after vaccination. Moreover, this method enabled the characterization of antigen specific CD8+ T cells that were previously undetected. Single-cell transcriptome profiling has the potential to allow for qualitative discrimination of cells, which could lead to novel insights on biological pathways involved in cellular responses. This approach could be further validated and allow for more informed decision making in preclinical and clinical settings.

## Introduction

T cells are a central component of the immune system; they are functionally heterogeneous and participate to different arms of the immune response. Full characterization of T-cell responses has proven to be a rather challenging task, mainly due to limitations of analysis approaches and techniques typically applied in the past. On one end, the bulk analysis of sampled material, through *omics* technologies like microarrays or next-generation sequencing, can only capture main trends in the signals. In these settings, the available information is limited to the averaged signals shared by the majority of cells under investigation, while more subtle, but potentially biologically meaningful, data on cell-to-cell differences are typically not generated (1). Other technology platforms like flow and mass cytometry provide information on a more limited panel of markers and, generally, do not fully resolve the complexity of the multifaceted T-cell functionality (2–6). More recently, high-throughput scRNAseq technologies (e.g. 10x Genomics Chromium) (7) have been developed for a deeper profiling of cellular immune responses to vaccination (8, 9). When investigating T-cell responses in vaccine studies, measuring the number of elicited antigen-specific T cells, or the activation of a limited number of markers may be an over-simplistic approach. This is especially true in early exploratory studies in which a better understanding of the mode of action of new vaccines or vaccine components is sought (2). In recent years, a number of technologies for single-cell transcriptomics have arisen (10). These technologies are generally based on microfluidic systems capable of handling nanoliter-scale samples that are used to isolate individual cells, which are then further processed to determine their transcriptional profiles. Single-cell measurements can provide a highly resolved picture of the underlying biology and allows the extraction of information that would be typically precluded, like for example the detection of cell subpopulations characterized by a specific transcriptional profile. In recent years, single-cell transcriptomics found numerous applications in a variety of fields, including cancer, embryonic development and immunology (11, 12).

Influenza virus is a major cause of respiratory tract infection in humans, causing recurring worldwide epidemics and representing one of the main causes of morbidity and mortality in the human population (13). Vaccination represents the most effective intervention against influenza disease and has been recommended worldwide in individuals from 6 months of age and in pregnant women in certain countries (14). Currently, the most widely used vaccines against influenza consist of inactivated vaccines, in which the two main surface antigens, hemagglutinin and neuraminidase are purified and enriched (15). To ensure effective protection, these vaccines include multiple virus strains and, in some cases, adjuvants (16). Recently, RNA-based vaccines have emerged as a promising alternative to conventional influenza vaccines, given their streamlined manufacturing and their proven ability to induce potent humoral and cell-mediated responses (17–21). Preclinical studies investigating the mode of action of a new self-amplifying mRNA (SAM) vaccine technology, recently showed that a SAM vaccine encoding for a H1N1 influenza hemagglutinin (SAM-H1) induced high functional antibody titers and high frequencies of cytotoxic and cytokine-polypositive CD8+ T cells, resulting in cross-protection against heterologous strains (18). Furthermore, comparative tests with a protein-based subunit vaccine suggested that the ability to induce cross-protection was dependent on the activation of CD4+ Th1 and CD8+ cytotoxic T cells (18). Antigens from RNA based vaccines follow the MHC class I presentation pathway to activate CD8+ T cells (22). In contrast, antigens from adjuvanted protein-based vaccines are taken up by antigen presenting cells, including monocytes, and follow the MHC class II presentation pathway to mainly activate CD4+ T cells (23), which could explain the paucity of cytotoxic CD8+ T cells elicited by adjuvanted protein-based vaccines. Such evidence suggested that SAM vaccines can induce broadly protective responses based, at least in part, to their ability to activate CD8+ T cells and highlighted the need to better characterize and further understand this particular component of the immune response to vaccination.

Cytotoxic CD8+ T cells are specialized to respond to intracellular pathogens and, as such, are at the frontline of the immune response to viruses like the influenza virus (24). Influenza-specific effector and memory CD8+ T cells following infection and/or vaccination are found in the secondary lymphoid organs and can persist in the lungs for several months (25, 26). One important goal in vaccinology research is to identify biomarkers that correlate with vaccine‐induced protection. Traditional methods used to measure the immunogenicity of vaccines do not represent the best option to predict their efficacy. The measurement of T‐cell functions is a way to assess vaccine efficacy and is currently limited to the measurements of intracellular cytokines after *in vitro* stimulation, phenotyping, cytotoxic assays, binding of tetramers to cell surface receptors and/or measurement of epitope immunoreactivity by flow cytometry (2, 27). These methods rely on a limited number of biomarkers measured simultaneously and do not provide detailed information regarding CD8+ T-cell heterogeneity. Even when purified, the CD8+ T-cell subsets are generally identified based on a relatively small number of biomarkers, compared to the abundant number of cell surface and intracellular proteins expressed (28). There is an essential need for new biomarkers and novel methods have been developed to assess vaccine‐associated immune parameters (29). Single-cell transcriptome analysis has been compared previously against microarray data in vaccine development. For example, single-cell transcriptome analysis of CD8+ T cells was used to discriminate between subsets of cells from animals receiving different vaccination regimens (30–32).

In the present study we used a BALB-c mouse model to characterize, in detail, the CD8+ T-cell responses to SAM-H1 candidate vaccine formulated with a cationic nanoemulsion (SAM-H1/CNE) and compared it with the response elicited by an MF59-adjuvanted monovalent influenza vaccine (aMIV). After antigen-specific CD8+ T-cell isolation and quantification, a microfluidic system was used to profile the transcriptional phenotype of single CD8+ T cells. We then applied a customized data analysis framework for the detection and characterization of cell subpopulations. Overall, our data showed that unique subsets of CD8+ T cells were differentially elicited by the two vaccines and confirmed the ability of SAM-based influenza vaccines to induce a stronger and more robust CD8+ T-cell response compared to an adjuvanted subunit vaccine. Furthermore, SAM-induced CD8+ T cells showed a remarkably higher transcriptional activity, which in specific subpopulations was characterized by the co-expression of multiple cytotoxic markers.

## Material and Methods

### RNA Synthesis and SAM-H1/CNE Formulation

RNA was prepared as previously reported (33, 34). Briefly, the H1 gene was amplified from the cDNA of the influenza virus A/California/7/2009 (H1N1) and cloned as *SalI* and *NotI* fragments into an optimized replicon construct. DNA plasmid encoding the H1 replicon was amplified in *Escherichia coli*, and purified using Plasmid Maxi kits (Qiagen). DNA was linearized immediately downstream of the 3’-end of the SAM sequence by endonuclease restriction digestion with *Pmel*. The linearized DNA Templates were purified by phenol/choloroform extraction and ethanol precipitation before being transcribed into RNA using MEGAscript T7 kit (LifeTechnologies). The RNA was capped using ScriptCap m7G Capping system (CellScript), purified by LiCl precipitation and suspended in nuclease-free water (LifeTechnology). SAM-H1 RNA integrity was evaluated on an 1% agarose-LE gel (Ambion). SAM-H1 RNA was formulated with CNE (35) prepared as previously reported (18, 35, 36). The RNA was added in a drop wise manner to an equal volume of CNE. The formulation was complexed for 45 min on ice and prepared fresh for each immunization. The particle size of the complex was measured by dynamic light scattering (Malvern) to 194 ± 76nM and injected within 2 h of preparation.

### H1N1 Antigen and MF59 Formulation

Live A/California/7/2009 (H1N1) viruses were injected into the allantoic cavity of embryonated chicken eggs and grown for 10 days, followed by harvesting, purification of H1N1 antigens, and storage at -80°C (18). On the day of immunization, aMIV was freshly prepared by formulating H1N1 antigens with 50% (vol:vol) oil-in-water MF59 nanoemulsion (37, 38). The size of MF59 was 160 nm and composed of polysorbate 80, sorbitan trioleate 85 and squalene (37). Immediately prior to *in vivo* administration, the vaccine formulations were characterized for osmolality (350 ± 60mOsm) and pH (7.0 ± 0.5), and for the degree of H1N1 absorption to MF59 using an SDS-PAGE gel.

### Animal Studies

BALB/c mice (Charles River Laboratories, Calco, Italy), aged 6–8 weeks, were immunized on study day 0 and 56 *via* intramuscular injection in the quadriceps muscle of each hind leg with 50 μl per leg of 15 μg SAM-H1/CNE, 3 μg aMIV, or sterile PBS (Sigma). Mice were sacrificed and their spleens harvested ten days and five to six weeks after the first immunization (D10P1 and W6P1, respectively), and ten days and six weeks after the second immunization (D10P2 and W6P2, respectively). The spleens were processed to a single-cell suspension, and red blood cells lysed by using a red blood cell lysis buffer (eBioscience) following manufacturer’s protocol.

### *Ex vivo* MHC-I HA_533-541_ Pentamer Staining and Sorting of Antigen-Specific Single CD8+ T Cells

For the detection of HA_533-541_-specific CD8+ T cells, splenocytes were stained with a live/dead-aqua (LifeTechnologies), anti-CD8 APC (BD Biosciences), linage markers (anti-CD14 FITC, anti-CD19 FITC, anti-CD335 FITC, anti-F4/80 FITC [BD Biosciences)] and a recombinant H-2K^d^-restricted MHC-I pentamer loaded with HA_533-541_ peptide and bound to PE-labeled streptavidin (50 µg/ml) (Proimmune) targeting the T cell receptor of HA_533-541_-specific CD8+ T cells (18). Pentamer positive (pent+) CD8+ T cells from vaccinated mice were single-cell sorted as lineage marker negative (CD14-, CD19-, CD335-, F4/80-), CD8+ and pent+ (**S1 Figure**). Cells were deposited into a 96-well plate (1 cell/well) containing nuclease-free water with 1mg/ml BSA (LifeTechnologies) and 1U/well RNasin (Fermentas). Single-cell sorting was performed using a FACS Aria III flow cytometer (Becton Dickinson).

### Cytotoxic Studies

The killing activity of HA_533-541_-specific CD8+ T cells induced by the different vaccines was determined *in vivo*. Splenocytes from naïve BALB/c mice were loaded with cognate 5 μM HA_533-541_ peptide (IYSTVASSL, JPT) or 10 μM HIV gag_199-207_ control peptide (AMQMLKETI, JPT) and after 1 h the cells were loaded with CFSE (LifeTechnology) or CMTMR (LifeTechnology), respectively. Splenocytes (10^6^) were adoptively transferred into the tail vein of recipient mice 9 days or 5 - 6 weeks after the first or second immunization with SAM-H1/CNE, aMIV or sterile PBS. Mice were sacrificed 20 h after the adoptive transfer, and splenocytes were stained with live/dead yellow (LifeTechnologies) and frequencies of CFSE+ or CMTMR+ cells were measured by flow cytometry on a LSRII SORP flow cytometer (Becton Dickinson). The target cell specific lysis was calculated as % specific lysis = [1-(naïve mice/vaccinated mice)]x100 (39).

### Multiplexing RT-qPCR of pent+CD8+ T Cells

cDNA was synthesized from the single cells, by adding 25 ng/μl oligo-dT primer, 50 ng/μl random hexamers, 1 mM dNTPs in nuclease-free water (LifeTechnologie). After 5 min pre-heating the plates to 65°C, 1.8x First Strand buffer, 0.01 M DiThioThreitol, 5U/μl Superscript III and 1U/μl RNAse Out added and run on the program: 25°C for 5 min, 50°C for 60 min, 15 min at 55°C and 70°C for 15 min. cDNAs were pre-amplified by adding nuclease-free water, 1.25x PreAmp Master Mix, 57 nM TaqMan primer and 16 nM probe (LifeTechonologies) in a multiplex reaction. 5 μl cDNA was mixed with 20 μl pre-amplification mix and run on a LC480II qPCR (Roche) using the program: 95°C for 10 min, 20 cycles at 95°C for 15 sec followed by 4 min at 60°C. The pre-amplified cDNA was diluted in 4 times TE buffer (LifeTechnologies) and loaded into the primed 96.96 Dynamic Arrays (Fluidigm), together with 1x loading reagent, nuclease-free water and 1.5x TaqMan PCR Master Mix (LifeTechnologies). The samples were loaded on the right side of the 96.96 Dynamic Array. 1x Assay Loading Reagent was mixed with the 3 μl of 96 different TaqMan gene expression assays (**S1 Table**) in a 96-well plate. Each assay was loaded into the left side of the 96.96 Dynamic Array. Manufacturer’s instructions on “96.96 Real-Time PCR workflow quick reference protocol” were followed.

### Transcriptome Data Analysis

Cycle threshold (Ct) values were calculated from the system’s software (BioMark Real-time PCR Analysis; Fluidigm). Transcript abundance values were calculated by subtracting the baseline Ct value from targets’ Ct values. Baseline Ct was inferred by computing the average of the highest Ct observed across all analyzed genes. Cells for which no *cd3* or *cd8* signal was detected and cells expressing either *cd4* or *cd19* were excluded from the analysis. Genes for which no signal was observed throughout the entire experiment were also filtered out, leading to a final working dataset of 86 markers and 1152 cells (**S2 Table**).

Single-cell gene expression profiles were explored using principal component analysis (PCA). Transcriptional patters induced by the two vaccines were compared by projecting cells from their original 86-gene space to the first two principal components. Most informative genes and gene co-expression patterns were characterized by combining, for each gene, the coefficients from the first two principal components using the following formula $s_{pc1,2}=\sqrt[{2}]{s_{pc1^{2}}+s_{pc2^{2}}}$ ; where *S*_pc1_ and *S_pc2_* indicate the gene loadings on the first and second component, respectively. The extent of separation of cell clusters in the PCA space was quantified using a clustering silhouette metric (40).

The quantification of gene expression across multiple cells (expression index) was computed as the product of the proportion of cells expressing a given gene and the average gene expression value in these cells as previously shown by McHeyzer et al. (41) (**S3 Table**). Finally, differences in the proportion of cells expressing a given gene, across different groups or conditions, were tested for statistical significance using a Fisher’s exact test followed by correction for multiple tests using the Benjamini-Hochberg procedure.

### Ethics Statement

All animal studies were ethically reviewed and carried out in accordance with European Directive 2010/63/EEC, the Italian legislation on the care and use of animals in experimentation (Legislative Decree 116/92), and the GSK policy on the Care, Welfare and Treatment of Animals.

## Results

### SAM-H1/CNE and aMIV Induce pent+CD8+ T Cells *In Vivo*

**Figure 1:**
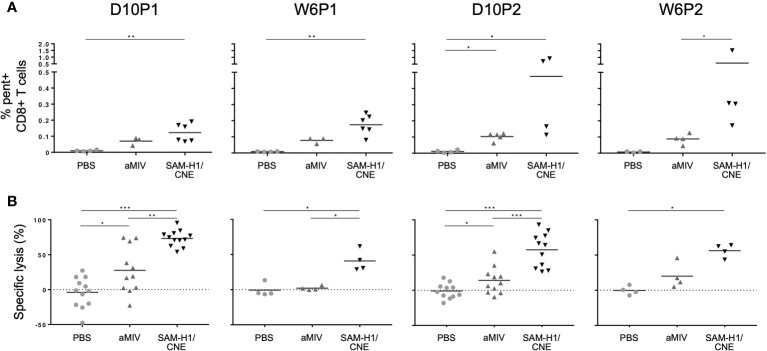
SAM-H1/CNE elicits pent+CD8+ T cells associated with cytotoxic activity *in vivo*. **(A)** Frequency of pent+CD8+ T cells in mice immunized with SAM-H1/CNE (black), aMIV (dark grey) or PBS (light grey). **(B)** *In vivo* lysis of adoptively transferred splenocytes loaded with HA_533-541_ or control HIV gag_199-207_ peptides. The graphs show the HA_533-541_-specific lysis of target cells in mice immunized with SAM-H1/CNE (black), aMIV (dark grey) or sterile PBS (light grey). *p ≤ 0.05, **p ≤ 0.01, ***p ≤ 0.001 (two-tailed Mann-Whitney U test).

We previously showed that SAM-H1/CNE and aMIV induced antigen-specific CD4+ T cells, but only SAM-H1/CNE induced CD8+ T cells (18), as measured by the production of key cytokines like IL-2, TNF and IFN-γ upon *in vitro* stimulation. In this study, we identified H1-specific CD8+ T cells using an HA_533-541_-pentamer (**Figure 1A**). HA_533-541_-pentamer positive (pent+CD8+) T cells were detected after a single immunization with either SAM-H1/CNE or aMIV. The frequency of pent+CD8+ T cells increased after a second dose of SAM-H1/CNE, provided 8 weeks later, while it remained unchanged after the second dose of aMIV. Pent+CD8+ T-cell frequencies in PBS immunized mice remained below detectable range at all time points. The data suggest that both vaccination strategies can induce a subset of HA_533-541_-specific CD8+ T cells, that were not previously detected using intracellular cytokine staining.

To explore if these pent+CD8+ T cells were functional, an *in vivo* experiment was performed to characterize the cytotoxic activity induced by each vaccine regimen 10 days after each immunization. Briefly, naïve splenocytes were loaded with HA_533-541_ peptide and CFSE, and adoptively transferred into previously vaccinated mice. The specific lysis of CFSE+ target cells, measured 20 h after adoptive transfer, was significantly higher in mice immunized with SAM-H1/CNE than in aMIV immunized mice, especially at D10P1 and D10P2, suggesting that SAM-H1/CNE does induce more cytotoxic CD8+ T cells compared to aMIV (**Figure 1B**). No specific lysis was detected in PBS control mice.

### Pent+CD8+ T Cell Elicited by SAM-H1/CNE and aMIV Segregate in *Cd62l-* and *Cd62l+* Subpopulations Characterized by Different Transcriptional Profiles

Pent+CD8+ T cells from pooled spleen of two mice from each of the immunization groups were single cell sorted and their mRNA transcribed into cDNA for further analyses. Ninety-six genes were selected based on their involvement in T-cell differentiation, tissue homing, survival, activation, cytotoxicity and regulation of immune responses (**S1 Table**) (28, 42–48). Cells with no expression signal for positive control genes (*Cd3*, *Cd8*) or with expression signal for negative control genes (*Cd4, Cd19*) were excluded from the analysis, for a total of 5 cells (**Table 1**). Furthermore, both cells and genes presenting no expression signals were also filtered out. Overall, eighty-six genes and 1152 single cells were retained for further analyses (**Table 1** and **S2 Table**).

**Table 1:** Number of pent+CD8+ T cells derived from a pool of two spleens from each immunization groups and at each timepoint profiled by RT-qPCR.

Time point	Number of pent+CD8+ T cells		Number of excluded cells	
	SAM-H1/CNE	aMIV	SAM-H1/CNE	aMIV
D10P1	106	166	2	–
W6P1	137	75	–	1
D10P2	175	172	–	1
W6P2	157	164	1	–

**Figure 2:**
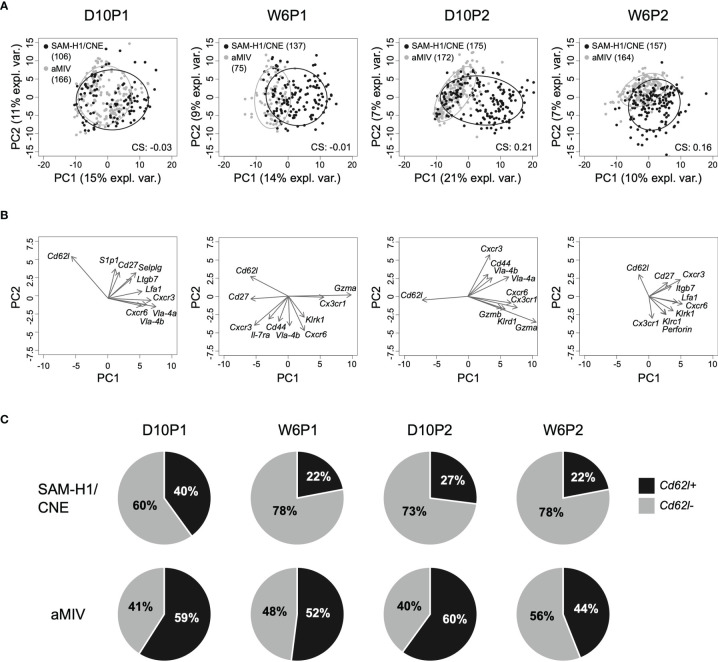
PCA of the transcriptome profiles of pent+CD8+ T cells. **(A)** Kinetics of individual pent+CD8+ T cells induced by SAM-H1/CNE (black) and aMIV (grey) represented on the first two principal components. CS: clustering score indicating the degree of cluster separation. **(B)** Loading plots representing the ten most informative genes across the different time points. **(C)** Relative proportion of *Cd62l+* and *Cd62l*- pent+CD8+ T cells across treatments and time points.

Differences in the transcriptional responses induced by the two vaccines in individual pent+CD8+ T cells were explored through PCA. This analysis revealed that the transcriptome profiles of individual pent+CD8+ T cells are quite heterogeneous, as suggested by the limited amount of variance explained by the first two PCA components across the different time points (PC1: 10-21% (min-max); PC2: 7-11% (min-max); **Figure 2A**). Despite the heterogeneity, however, it was still possible to appreciate that a substantial proportion of cells induced by SAM-H1/CNE were transcriptionally different from cells induced by aMIV. This is reflected by the extent of separation between the two cell clusters, which was most evident after the second immunization (D10P2 clustering score: 0.21; **Figure 2A**). Furthermore, cells collected following the first immunization showed substantial overlap (D10P2 clustering score ≈ 0; **Figure 2A**), suggesting that the second immunization expanded pent+CD8+ T-cell subpopulations that were not detectable after the first immunization.

To get a better understanding of which genes were driving the scattering of cells on the first two PCA components, the 10 most informative genes were selected, and represented in the form of loadings plots (**Figure 2B**). We observed that the shift of cells along the first principal component was mainly driven by the higher expression of a subset of genes (*Gzma*, *Gzmb*, *Klrd1*, *Cxcr6* and *Cx3cr1*) in the SAM-H1/CNE group, coupled with a lower expression of *Cd62l* in the same group (**S3 Table**). Interestingly, *Cd62l* appeared among the 10 most informative genes in all the analyzed time points. *Cd62l*, a gene encoding for a cell-adhesion protein, is a key molecule involved in cell trafficking to secondary lymphoid organs and has been reported to distinguish between naïve and effector cells (28) and short lived effector T cells (31). SAM-H1/CNE induced 60 - 78% of *Cd62l-* pent+CD8+ T cells, whereas aMIV induced a more balanced *Cd62l-* to *Cd62l+* ratio (40 - 56% of *Cd62l-* cells; **Figure 2C**).

**Figure 3:**
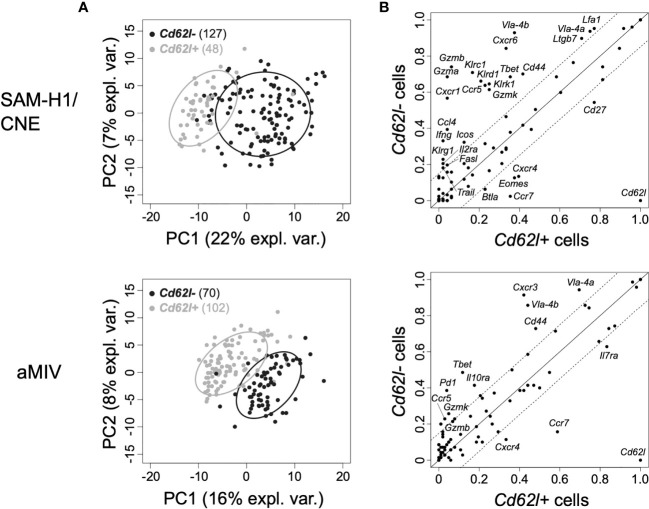
PCA of the transcriptome profiles of *Cd62l+* and *Cd62l-* pent+CD8+ T cells at D10P2. **(A)** Individual *Cd62l+* (black) and *Cd62l-* (grey) pent+CD8+ T cells representation on the first two principal components space for SAM-H1/CNE (top panel) and aMIV (bottom panel) at D10P2. **(B)** Proportion of *Cd62l+ vs Cd62l-* pent+CD8+ T cells expressing each individual gene in response to SAM-H1/CNE (top panel) or aMIV (bottom panel). Solid lines indicate equality (y=x), while the area within the dotted lines represents the ±15% tolerance interval (≤ 15% difference between *Cd62l+* and *Cd62l-* cell populations).

We further compared the transcriptional profiles of *Cd62l-* and *Cd62l+* pent+CD8+ T cells induced by SAM-H1/CNE and aMIV to gather insights on the functionality of these populations. The PCA analysis confirmed the relevance of *Cd62l* in defining functionally different CD8+ T cells as we observed an almost complete segregation of the two cell populations (**Figure 3A**), reflecting a substantially different transcriptional phenotype. *Cd62l-* pent+CD8+ T cells were generally more transcriptionally active than their *Cd62l+* counterparts, with 22 *vs* 6 and 10 *vs* 4 upregulated genes within the SAM-H1/CNE and aMIV groups, respectively (**Figure 3B**).

### Transcriptional Differences Between SAM-H1/CNE- and aMIV-Induced pent+CD8+ T Cells Are Restricted to the *Cd62l-* Subpopulation

**Figure 4:**
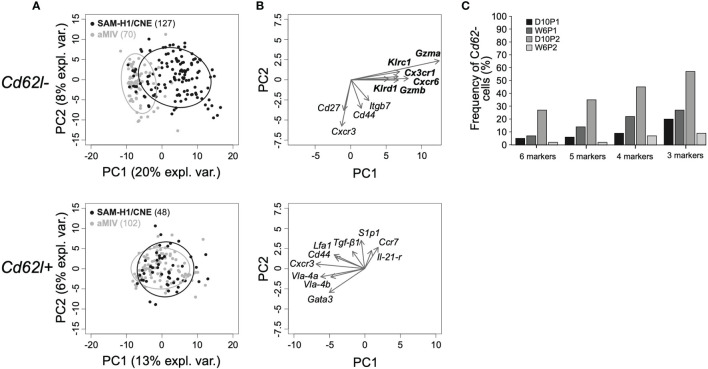
Genes characterizing *Cd62l-* pent+CD8 T cells induced by SAM-H1/CNE at D10P2. **(A)** Representation of individual *Cd62l*- (top panel) and *Cd62l*+ (bottom panel) pent+CD8+ T cells induced by SAM-H1/CNE (black) or aMIV (grey) on the first two principal components for the D10P2 time point. **(B)** Loading plots representing the ten most informative genes. **(C)** Co-expression analysis of genes driving the segregation between *Cd62l-* pent+CD8+ T cells induced by SAM-H1/CNE- and aMIV. 6 markers: *Klrc1*, *Gzma*, *Cx3cr1*, *Cxcr6*, *Gzmb* and *Klrd1*. 5 markers: *Klrc1*, *Gzma*, *Cxcr6*, *Gzmb* and *Klrd1*. 4 markers: *Klrc1*, *Gzma*, *Cxcr6* and *Gzmb*. 3 markers: *Gzma*, *Cxcr6* and *Gzmb*.

Next, we focused on the comparison of *Cd62l-* and *Cd62l+* pent+CD8+ T-cell subpopulations across the two treatments. The projection on the principal component space (first two components) of individual *Cd62l-* and *Cd62l+* pent+CD8+ T cells in response to either SAM-H1/CNE or aMIV showed that *Cd62l-* pent+CD8+ T cells segregated into different clusters, reflecting distinct transcriptional phenotypes. Conversely, *Cd62l+* pent+CD8+ T cells did not segregate and showed substantial overlap (**Figure 4A**).

Loadings analysis data revealed that the shift of *Cd62l-* pent+CD8+ T cells along the first principal component induced by SAM-H1/CNE was primarily driven by the upregulation of 6 transcripts, including genes encoding for cytotoxic (*Gzma*, *Gzmb, Klrc1*, *Klrd1*), proinflammatory (*Cxcr6*), and homing (*Cx3cr1*) markers (**Figure 4B** and **S2 Figure**). The small relative angles defined by the arrows representing the loadings for these six genes (**Figure 4B**, upper panel) indicate that these may be co-regulated. To substantiate this hypothesis, the relative frequencies of cells co-expressing these genes were computed. At D10P2, 27% of the SAM-H1/CNE-induced *Cd62l-* pent+CD8+ T cells were positive for all six markers (**Figure 4C**). Similarly, 35%, 45% and 57% of SAM-H1/CNE-induced pent+CD8+ T cells were co-expressing five (*Klrc1*, *Gzma*, *Cxcr6*, *Gzmb*, *Klrd1*), four (*Klrc1*, *Gzma*, *Cxcr6, Gzmb*) or three (*Gzma*, *Cxcr6*, *Gzmb*) genes, respectively. At the other time points, the level of co-expression of these markers was lower, even though a similar trend was observed. Furthermore, the transcript abundance of *Klrg1*, *Blimp1* and *Il-2rα*, genes, whose products were reported to induce differentiation of activated CD8+ T cells into cytotoxic T cells (48), was generally higher in *Cd62l*- than in *Cd62l+* pent+CD8+ T cells, especially within the SAM-H1/CNE-group (**S2 Figure**).

**Figure 5:**
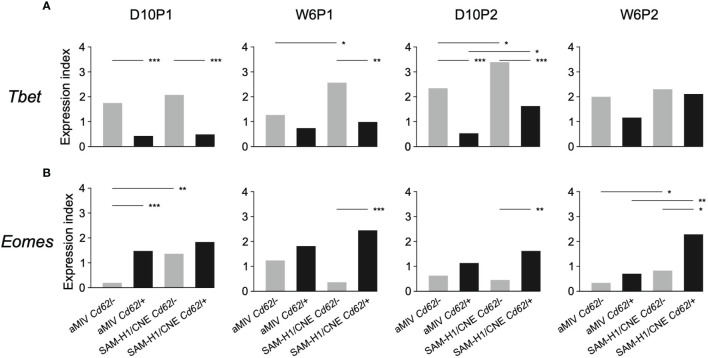
Kinetics of **(A)** *Tbet vs* **(B)** *Eomes* gene expression in *Cd62l-* and *Cd62l*+ pent+CD8+ T-cell populations. Differences between groups were tested by comparing the frequencies of cells expressing each specific marker through the Fisher’s exact test. *p*-values were corrected for multiple testing using the Benjamini-Hochberg procedure. *p ≤ 0.05, **p ≤ 0.01, ***p ≤ 0.001.

*Tbet* and *Eomes* are two master regulators of T-cell development and their coordinated activation has been proposed to regulate the formation of effector CD8+ T-cell functions (49). Within both SAM-H1/CNE and aMIV groups, *Tbet* expression was generally higher in *Cd62l-* pent+CD8+ T cells compared to their *Cd62l+* counterpart, with differences being highly significant at early time points after immunization (**Figure 5A**, D10p1 and D10P2; adjusted Fisher’s exact test *p*-value ≤ 0.001). An opposite trend, albeit less consistent, was observed for *Eomes*, whose expression was generally higher in *Cd62l+* pent+CD8+ T cells (**Figure 5B**). These evidences are in agreement with the former observation of *Cd62l-* pent+CD8+ T cells being characterized by a cytotoxic, effector phenotype.

Overall, these findings indicate that SAM-H1/CNE shifted the CD8+ T-cell response towards a more effector-cytotoxic profile by specifically modulating the response of *Cd62l-* pent+CD8+ T cells.

### SAM-H1/CNE Induces pent+CD8+ T Effector and Effector Memory Cells With a Cytotoxic Transcriptional Profile

**Figure 6:**
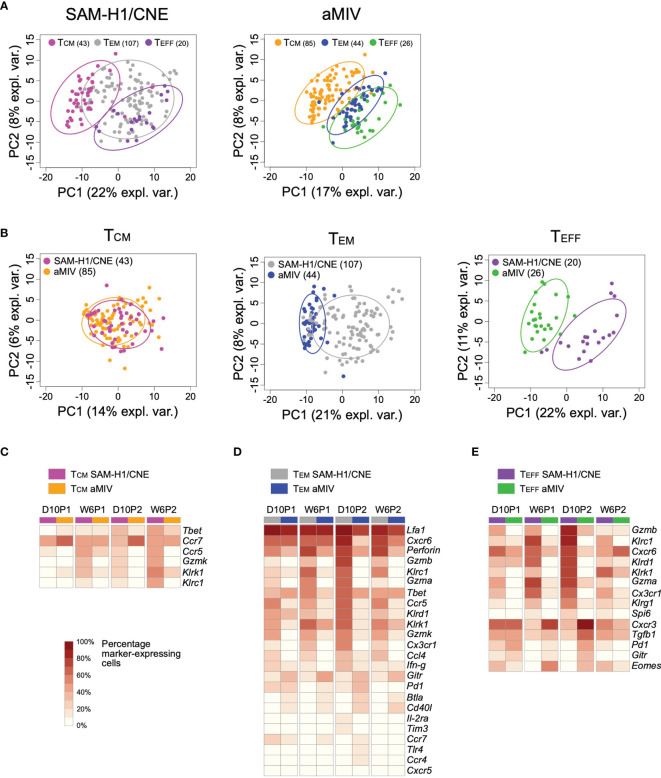
Transcriptome profiles of pent+CD8+ T_CM_, T_EM_ and T_EFF_ cell subpopulations induced by SAM-H1/CNE and aMIV at D10P2. Representation of individual cells on the first two principal components for the T_CM_, T_EM_ and T_EFF_ cells in response to either SAM-H1/CNE or aMIV. **(A)** Comparison of the three cell populations. **(B)** Comparison of each specific cell population. **(C–E)** Heatmap representing the percentage of cells expressing each specific gene within the T_CM_, T_EM_ and T_EFF_ cells. Only gene showing a significant difference (adjusted Fisher’s exact test *p*-value ≤ 0.05) between SAM-H1/CNE and aMIV, for the D10P2 time point, are represented. Differences between groups were tested by comparing the frequencies of cells expressing each specific marker through the Fisher’s exact test. *p*-values were corrected for multiple testing using the Benjamini-Hochberg procedure.

The combinatorial expression of *Il-7rα* and *Cd62l* discriminates among three T-cell differentiation states: effector (T_EFF_; *Il-7rα-Cd62l-*), effector memory (T_EM_; *Il-7rα+Cd62l-*) and central memory (T_CM_; *Il-7rα+Cd62l+*) T cells. These three stages are characterized by various degrees of proliferative and cytotoxic potentials (50). PCA analysis of D10P2 responses showed that, regardless of the immunization regimen, these T-cell subsets displayed some degree of separation on the first two principal components space, highlighting different transcriptional profiles (**Figure 6A**).

Head-to-head comparison of each individual T-cell memory subset induced by SAM-H1/CNE and aMIV highlighted an overall similar T_CM_ transcriptional response (**Figures 6B, C**), while T_EFF_ and T_EM_ subsets were characterized by different transcriptional profiles (**Figures 6B, D, E**). A higher proportion of T_EFF_ and T_EM_ cells upregulated various activation, cytotoxic, and pro-inflammatory genes *(Cxcr6, Gzmb, Gzma, Gzmk, Inf-γ, Klrc1, Klrd, Klrk, Perforin*) in response to SAM-H1/CNE compared to aMIV (**Figures 6D, E**), suggesting that SAM-H1/CNE increased the cytotoxic activity of CD8+ T cells. In contrast, genes associated with exhaustion, survival, and homeostasis (*Btla, Cd40l,Gitr, Pd1*) were more frequently upregulated in aMIV-induced cells at D10P2 (**Figures 6D, E**) (51–55).

Overall, these findings confirm the cytotoxic nature of pent+CD8+ T cells induced by SAM-H1/CNE, in agreement with previous observations (18), and suggest that CD8+ T cells elicited by aMIV contain a subpopulation of activated cells that exhibited a regulatory and possibly exhausted profile in mice.

## Discussion

In recent years, single-cell technologies have enabled the high-resolution assessment of cellular heterogeneity in immune responses (56–58). Single-cell transcriptome analysis has been applied to explore the biological functions of CD8+ T cells in different systems (58, 59), displaying high heterogeneity that could be explained by different levels of cellular activation (60). This kind of approach also found extensive applicability in vaccine studies, where they expanded the current understanding of the mechanisms underlying CD8+ T-cell responses to HIV (30) and dengue vaccines (8). In this study, we used a high throughput quantitative PCR system to characterize the transcriptome profiles of individual antigen specific CD8+ T cells in response to either an mRNA (SAM H1/CNE) or an adjuvanted subunit (aMIV) influenza vaccine. Our single-cell multi-facetted approach revealed that SAM-H1/CNE induced mainly CD8+ T_EFF_ and T_EM_ cells characterized by the upregulated expression of various activation, cytotoxic, and pro-inflammatory genes like *Gzmb, Gzma, Gzmk, Perforin, Inf-γ, Klrc1, Klrd1, Klrk1* and *Cxcr6.* A previous study showed that single-cell gene expression analysis of human CD8+ T cells 14 days post Dengue vaccination revealed a cluster of cells enriched by effector genes such as *Gzma*, *Gzmb* and P*rf1* (8). Additionally, scRNA-seq analysis of murine virus-specific CD8+ T cells from both acute and chronic lymphocytic choriomeningitis virus infections exhibited diffuse expression of *Gzmb* and *Cxcr6* (58). Moreover, single-cell transcriptomic analysis of tetramer+ CD8+ T cells from splenocytes of murine cytomegalovirus infected mice exhibited high expression of *Klrg1* and *Cx3cr1* transcripts compared to the same cells isolated from the gut (59).

In this study, we characterized the response of individual antigen-specific CD8+ T cells by complementing an initial flow cytometry cell sorting with a high-throughput, single-cell qPCR assay capable of monitoring the modulation of up to 96 different markers. Upon identification of a smaller panel of key markers, flow or mass cytometry which allow for the analysis of protein expression of 30-40 markers on a single-cell level (55, 61), could be applied in a second step to confirm the CD8+ T cell gene signature. Cytotoxic CD8+ T cells have usually been identified by measuring the expression of TNF, IFN-γ, IL-2, and CD107 degranulation after *in vitro* stimulation (18, 19), arguably resulting in a limited ability to detect and characterize CD8+ T-cell subsets. In fact, pentamer- rather than intracellular cytokine staining enabled us, for the first time, to detect H1-specific CD8+ T cells induced by aMIV, an adjuvanted protein-based vaccine. Despite this innovative component, however, our approach was limited by the fact that the pentamer targeted a single H1-specific epitope and likely captured only a fraction of the total H1-specific CD8+ T cells. Nonetheless, the epitope targeted (HA_533-541_) was reported to be immunodominant in several studies (62, 63). Principal component analysis of individual pent+CD8+ T-cell transcriptome profiles revealed an extensive similarity between the responses elicited by the two vaccines after the first dose. At the D10P2 time point, however, a substantial proportion of pent+CD8+ T cells induced by SAM-H1/CNE was characterized by a unique transcriptional phenotype, which was not elicited by aMIV. Based on this observation, we decided to focus our analysis on the D10P2 responses to further characterize the differences between the two vaccines.

*Cd62l*, coding for an adhesion/homing receptor that is usually expressed on naïve and central memory CD8+ T cells (28), was one of the most informative genes distinguishing between SAM-H1/CNE- and aMIV-induced pent+CD8+ T cells. The proportion of *Cd62l-* pent+CD8+ T cells was consistently higher in response to SAM-H1/CNE compared to aMIV (73% and 40%, respectively). *Cd62l-* pent+CD8+ T cells elicited by SAM-H1/CNE expressed a panel of genes (*Klrg1*, *Blimp1*, and *Il-2rα*) previously reported to be associated to cytotoxic CD8+ T cells (64–68) addition, these CD8+ T cells were characterized by a higher expression of genes encoding for cytotoxic mediators (GZMA, GZMB, KLRD1 and KLRC1) and homing receptors (CXCR6 and CX3CR1). Lung resident memory CD8 T cells (T_RM_), characterized as CD62L- and CXCR6+ (69–71), have been previously associated with immunity to respiratory pathogens like influenza and *Mycobacterium tuberculosis* (72, 73). At D10P2, 57% of the *Cd62l-* pent+CD8+ T cells co-expressed *Gzma*, *Gzmb* and *Cxcr6*, suggesting that 2 doses of SAM-H1/CNE may induce pent+CD8+ T cells with high cytotoxic effector and tissue-resident properties. mRNA vaccines have recently revealed to induce immunity against various pathogens (74–76). Our data are aligned with previous work which showed that an influenza based mRNA vaccine induced activation of INF-γ producing CD8+ T cells 12 days after a single immunization in mice (77). Moreover, recent publications provided evidence that mRNA based vaccine against SARS-CoV-2 induced a CD8+ T cell response (78, 79).

The combinatorial expression of *Cd62l* and *Il-7rα* can discriminate between CD8+ T_EFF_, T_CM_, and T_EM_ cells, characterized by divergent differentiation stage and cytotoxic potential (31, 80–82). We then characterized these three subpopulations in response to SAM-H1/CNE or aMIV. Pent+CD8+ T_CM_ cells elicited by the two vaccines were substantially similar. While Pent+CD8+ T_EFF_ and T_EM_ cells were transcriptionally different, with genes encoding for effector/cytotoxic functions (*Gzma*, *Gzmb, Gzmk*, *Perforin*) being more predominant among SAM-H1/CNE induced cells, a few markers were preferentially activated in response to aMIV. These included *Gitr* and *Cd40l*, two members of the tumor necrosis factor superfamily, and two immunomodulatory genes *Pd1* and *Btla*, suggesting that aMIV may induce Pent+CD8+ T_EFF_ and T_EM_ cells with more regulatory and helper functions (52, 55, 83).

These results suggest that SAM is a promising technology for inducing protection against pathogens that are not effectively neutralized by antibodies alone. Adjuvanted proteins have been on the market for decades but have been directed mostly towards the induction of neutralizing antibodies, and vaccination against new and emerging viruses like SARS-CoV-2 may not be sufficient to be covered by neutralization antibodies alone. Studies have shown that infections with SARS in patients generates short lived B-cells and neutralizing antibodies prone to antigen escape (1-2 years of coverage) with a high chance of re-infection, meanwhile generating long lasting T-cell memory in surviving patients (6-17 years of coverage) (84–86). It was further shown that these T cells can recognize antigens that B-cells do not recognize (87). At this time, two mRNA and two adenovector-based Covid-19 vaccines have received emergency authorized use and one adjuvanted protein vaccines has shown potential in Phase 3 clinical trials. If vaccines could be directed early on for the immune response in the target group of interest, a more efficient vaccine development could be implemented for both present and future emerging diseases.

Most currently available influenza vaccines are based on hemagglutinin and neuraminidase (88), two main viral surface constituents. The ability to target also influenza virus internal proteins like nucleoprotein and matrix protein, however, holds the potential of an increased vaccines’ breath of coverage and the induction of a longer-lasting immunity (89, 90). To this end, the ability to quickly design and assort different antigens offered by mRNA vaccines will be highly advantageous. Protection mediated by non-surface exposed proteins, however, relies on cell-mediated immunity rather than humoral immunity. For this reason, approaches that allow for the identification and characterization of relevant immune cells will help the development of next-generations vaccines.

In the present study, we characterized the transcriptional profiles of vaccine-induced HA_533-541_-specific CD8+ T cells, at the single-cell level, and identified unique *Cd62l*- Pent+CD8+ T_EFF_ and T_EM_ cell subsets that were differentially elicited by two immunization regimes in BALB/c mice. Extending these findings to intracellular influenza antigens and other species, including humans, may provide insightful understanding of vaccines’ mechanisms of action. Our observations support the integration of single-cell gene expression analysis into the characterization of CD8+ T-cell responses to vaccination, since the magnitude of antigen-specific cytokine-producing CD8+ T cells alone may not be an adequate correlate of protection (25, 91). In conclusion, this study provided evidence to support the use of single-cell transcriptomics as a complementary technology to more frequently adopted technologies, such as mass and flow cytometry. Single-cell transcriptomic profiling allows for qualitative discrimination of cells and the ability to find patterns of co-expressed genes that provide insights on biological pathways involved in cellular responses. The insights obtained in this study are a result of simultaneously analyzing numerous genes in parallel at the single-cell level and provide a more accurate view of cellular phenotypes. The application of these methods to CD8+ T cells and other immune cell subsets will add clarity to the underlying molecular mechanisms controlling cellular responses to vaccination.

## Data Availability Statement

The raw data supporting the conclusions of this article will be made available by the authors, without undue reservation.

## Ethics Statement

The animal study was reviewed and approved by The Italian Ministry of Health and the Italian legislation on the care and use of animals in experimentation (Legislative Decree 116/ 92).

## Author Contributions

SB and SN conceived the study. ES conceived the data analysis approach. CS, RK, ST, and TSM performed the experiments. FB and DMa analysed the data. TSM, FB, ES, and SB wrote the manuscript. All authors reviewed and approved the manuscript.

## Funding

TSM, RK, FB, and DMa participated in a post graduate fellowship program at GSK. TSM and RK fellowships were funded by Marie-Curie ITN grant HOMIN FP7-PEOPLE- 2012-ITN (626283) and FP7-MC-IEF (274125), respectively.

## Conflict of Interest

This work was undertaken at the request of and sponsored by GlaxoSmithKline Biologicals SA. GSK directed all aspects of this study. CS, ST, SN, RK, ES and SB are all employees of the GSK group of companies. SN, DMe, ES, SB hold GSK shares. MF59 is a trademark of Novartis AG.

## Publisher’s Note

All claims expressed in this article are solely those of the authors and do not necessarily represent those of their affiliated organizations, or those of the publisher, the editors and the reviewers. Any product that may be evaluated in this article, or claim that may be made by its manufacturer, is not guaranteed or endorsed by the publisher.

## Abbreviations

SAM, self-amplifying mRNA; CNE, cationic nanoemulsion; aMIV, MF59-adjuvanted monovalent influenza vaccine; H1N1, influenza virus A/California/7/2009; D10P1, ten days after the first immunization; W6P1, five to six weeks after the first immunization; D10P2, ten days after the second immunization; W6P2, six weeks after the second immunization; Pent+, Pentamer positive; Ct, Cycle threshold; PCA, principal component analysis; Pent+CD8+, HA_533-541_-pentamer positive; TEFF, T-cell differentiation effector state; TEM, T-cell differentiation effector memory state; TECM, T-cell differentiation central memory state.
