# Supplementary material for: Single-Cell Analysis of Antigen-Specific CD8+ T-Cell Transcripts Reveals Profiles Specific to mRNA or Adjuvanted Protein Vaccines
Source: Front Immunol. 2021 Oct 29;12:757151. doi: 10.3389/fimmu.2021.757151 (PMC8586650; doi:10.3389/fimmu.2021.757151)

S1 Fig: Gating strategy for sorting single pent+CD8+ T cells.

Splenocytes were stained with a live/dead-aqua, anti-CD8 APC, linage markers (anti-CD14 FITC, anti-CD19 FITC, anti-CD335 FITC, anti-F4/80 FITC) and a recombinant H-2K d-restricted MHC-I pentamer loaded with HA 533-541 peptide and bound to PE-labeled streptavidin. Pentamer positive (pent+) CD8+ T cells were single-cell sorted as lineage marker negative (CD14-, CD19-, CD335-, F4/80-), CD8+ and pent+.

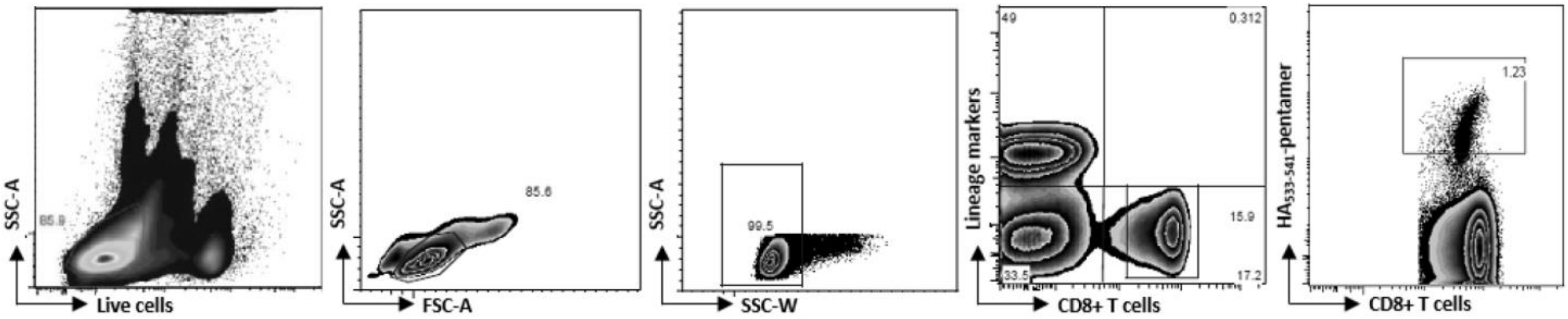

Supplement: Supplementary file 1 [file DataSheet_1.pdf]
