# Supplementary material for: Single-Cell Analysis of Antigen-Specific CD8+ T-Cell Transcripts Reveals Profiles Specific to mRNA or Adjuvanted Protein Vaccines
Source: Front Immunol. 2021 Oct 29;12:757151. doi: 10.3389/fimmu.2021.757151 (PMC8586650; doi:10.3389/fimmu.2021.757151)
Supplement: Supplementary file 2 [file DataSheet_2.pdf]

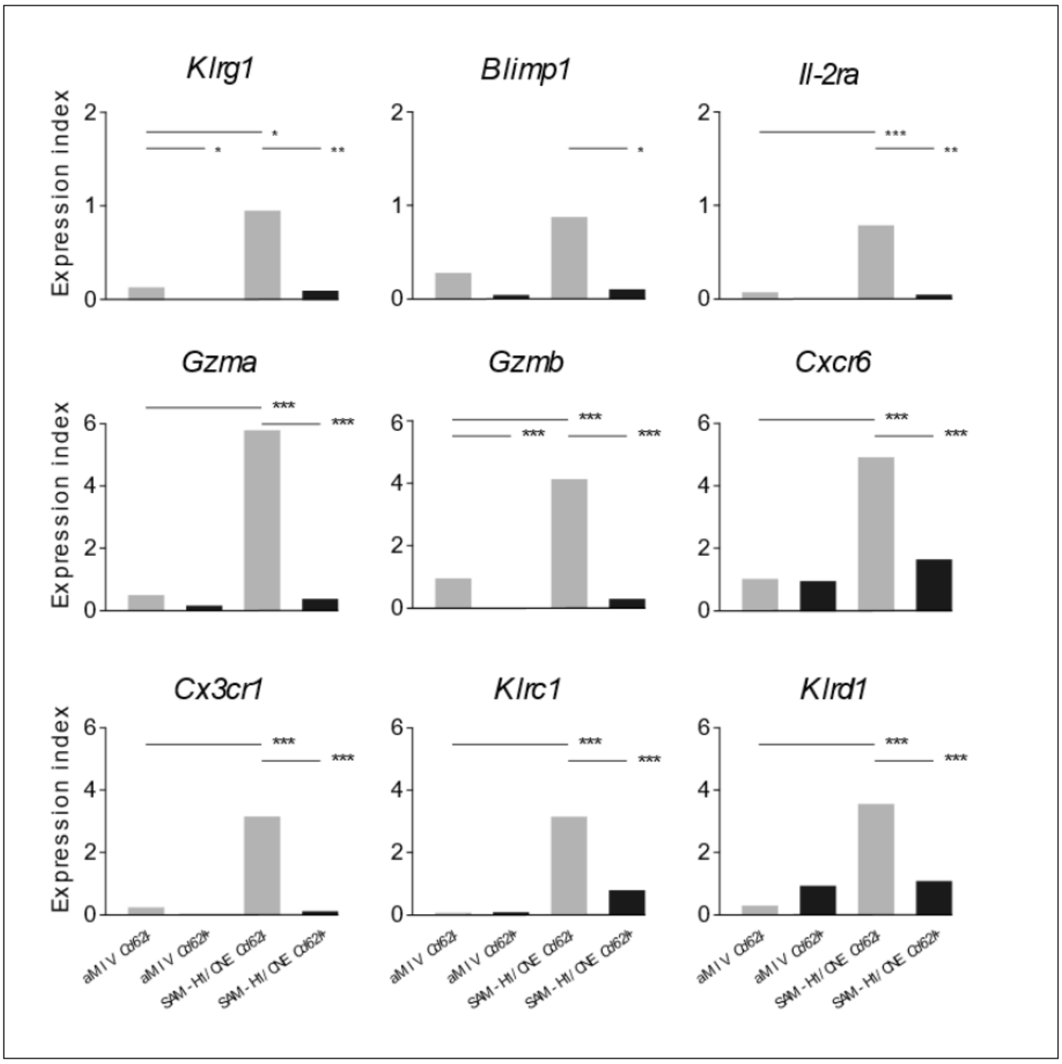

S2 Fig: Expression index of *Klrg1*, *Blimp1*, *Il-2rα*, *Gzma*, *Gzmb*, *Cxcr6*, *cx3cr1*, *Klrc1* and *Klrd1* in *Cd62l*+ and *Cd62l*- pent+CD8+ T cell compartments. Kinetics of *Klrg1*, *Blimp1*, *Il-2rα*, *Gzma*, *Gzmb*, *Cxcr6*, Cx3CR1, *Klrc1* and *Klrd1* expression in *Cd62l*- and *Cd62l*+ pent+CD8+ T-cell populations. Differences between groups were tested by comparing the frequencies of cells expressing each specific marker through the Fisher's exact test. *p*-values were corrected for multiple testing using the Benjamini-Hochberg procedure. * $p \leq 0.05$, ** $p \leq 0.01$, *** $p \leq 0.001$.
