# Supplementary material for: Single-Cell Analysis of Antigen-Specific CD8+ T-Cell Transcripts Reveals Profiles Specific to mRNA or Adjuvanted Protein Vaccines
Source: Front Immunol. 2021 Oct 29;12:757151. doi: 10.3389/fimmu.2021.757151 (PMC8586650; doi:10.3389/fimmu.2021.757151)
Supplement: Supplementary file 3 [file Table_1.pdf]

S1 Table: List of 96 different TaqMan gene expression assays.

Gene Symbol	Function in cells	Location	TaqMan	bp
Ahr	Regulation	TF / Nucleus	Mm00478932_m1	61
Bcl3	Priming	TF / Nucleus	Mm00504306_m1	83
Bcl6	Memory (Tcm)	TF / Nucleus	Mm00477633_m1	112
Bcl11b	Development	TF / Nucleus	Mm00480516_m1	152
Blimp1	SLEC, homeostasis	TF / Nucleus	Mm00476128_m1	72
Camkiv	Regulation	TF / Nucleus	Mm01135329_m1	65
Eomes	MPEC	TF / Nucleus	Mm01351985_m1	58
Foxo3a	Regulation	TF / Nucleus	Mm01185722_m1	71
Foxp3	Treg	TF / Nucleus	Mm00475162_m1	74
Gata3	Homeostasis and activation	TF / Nucleus	Mm00484683_m1	57
Irf4	Expansion and maintenance	TF / Nucleus	Mm00516431_m1	62
Mki67	Proliferation	TF / Nucleus	Mm01278616_m1	95
Nf-kβ1	Maintenance	TF / Nucleus	Mm00476379_m1	83
Relb	Maintenance	TF / Nucleus	Mm00485664_m1	58
Rorc	Tc17 generation	TF / Nucleus	Mm01261022_m1	54
Sstat5a	Memory homeostasis	TF / Nucleus	Mm00839861_m1	69
Ttbet	SLEC	TF / Nucleus	Mm00450960_m1	69
Bim	Regulation	Cytoplasm	Mm00437796_m1	64
Gcn2	Regulation	Cytoplasm	Mm00469222_m1	102
Gzma	Cytotoxicity - serine protease A	Cytoplasm	Mm00439191_m1	73
Gzmb	Cytotoxicity - serine protease B	Cytoplasm	Mm00442834_m1	95
Gzmk	Cytotoxicity - serine protease K	Cytoplasm	Mm00492530_m1	86
itk	Development	Cytoplasm	Mm00439861_m1	70
mapk8	Transcription regulation	Cytoplasm	Mm00489514_m1	99
perforin	Pore forming cytolytic protein	Cytoplasm	Mm00812512_m1	95
spi6	Inhibit cytotoxic apoptosis	Cytoplasm	Mm00777163_m1	125
traf2	Homeostasis	cytoplasm	Mm00801978_m1	75
Was	Activation of naive $CD8^{+}$ T cells	Cytoplasm	Mm00494167_m1	137
Klrc1	Prevent apoptosis, preserve $CD8^{+}$ T-cells	Plasma membrane	Mm01183333_m1	79
Klrd1	Regulate antiviral $CD8^{+}$ T cells	Plasma membrane	Mm00495182_m1	62
Klrg1	SLEC	Plasma membrane	Mm00516879_m1	81
Klrk1	Activation of cytotoxic $CD8^{+}$ T cells	Plasma membrane	Mm00473603_m1	124
Il-1r	Enhances antigen-driven CD8 T cell responses	Plasma membrane	Mm00434237_m1	63
Il-2rα	Stimulation - differentiation into memory	Plasma membrane	Mm01340213_m1	83
Il-2rγ	Stimulation - differentiation into memory	Plasma membrane	Mm00442885_m1	65
Il-7rα	MPEC seperation of activated Tcells during infection	Plasma membrane	Mm00434295_m1	100
Il-10rα	Insulating of cells from inflammatory signals	Plasma membrane	Mm00434151_m1	66
Il-12r-β1	Essential for resistance to intracellular pathogens	Plasma membrane	Mm00434189_m1	60
Il-21r	Long-term maintenance and functionality	Plasma membrane	Mm00600319_m1	65
Ccr4	Skin/lung homing	Plasma membrane	Mm00438271_m1	65
Ccr5	Memory, lung homing during viral infection	Plasma membrane	Mm01216171_m1	78
Ccr6	Homing for T_{em} cells - mucosal	Plasma membrane	Mm01323931_m1	78
Ccr7	Lymphatic organ homing	Plasma membrane	Mm01301785_m1	78
Ccr10	Skin/lung homing	Plasma membrane	Mm01292449_m1	72
Cx3cr1	Antiviral homing	Plasma membrane	Mm00438354_m1	70
Cxcr3	Inflammatory homing	Plasma membrane	Mm00438259_m1	58
Cxcr4	Bone marrow homing (Tcm)	Plasma membrane	Mm01292123_m1	99
Cxcr5	Entry to follicular areas	Plasma membrane	Mm00432086_m1	126
Cxcr6	Entry to inflammmed tissue	Plasma membrane	Mm00472858_m1	90
Cd3	Part of T cell receptor	Plasma membrane	Mm00599683_m1	110
Cd4	Co-stimulatory	Plasma membrane	Mm00442754_m1	78
Cd8	Co-stimulatory	Plasma membrane	Mm01182108_m1	67
Lfa1	Co-stimulatory migration and activation	Plasma membrane	Mm00801807_m1	119
Cd19	B-lymphocyte antigen	Plasma membrane	Mm00515420_m1	69
Cd27	Co-stimulatory maintenance and survival	Plasma membrane / TNFRSF	Mm01185212_g1	87
Cd28	Co-stimulatory antiviral immunity	Plasma membrane	Mm00483137_m1	68
Vla4b	VLA4 complex, directed into inflammed tissue	Plasma membrane	Mm01253230_m1	75
Cd44	Antigen mature activated cells	Plasma membrane	Mm01277163_m1	83
Vla4a	VLA4 complex, directed into inflammed tissue	Plasma membrane	Mm00439770_m1	69
Cd62l	Stimulation and entry into LN	Plasma membrane	Mm00441291_m1	101
Cd69	Homing	Plasma membrane	Mm01183378_m1	76
Itgae	Lung homing after viral infection w. CD69	Plasma membrane	Mm00434443_m1	69
Itgb7	Gut homing	Plasma membrane	Mm00442916_m1	79
Lamp1	Degranulation	Plasma membrane	Mm00495262_m1	76
Ox40	Co-stimulatory recall memory responses	Plasma membrane / TNFRSF	Mm00442037_g1	64
41bb	Co-stimulatory survival	Plasma membrane / TNFRSF	Mm00441899_m1	71
Ctla4	Regulates effector functions of $CD8^{+}$ T cells	Plasma membrane	Mm00486849_m1	71
Cd160	Co-inhibitory function	Plasma membrane	Mm00444462_m1	99

Selplg	inflammation/resting cell homing to lympnoid	Plasma membrane	Mm01204601_m1	91
Btla	co-inhibitory molecules (cancer)	Plasma membrane	Mm00616981_m1	71
Icos	Co-stimulatory activation	Plasma membrane	Mm00497600_m1	65
Pd1	Exhaustion, supression and terminally differentially	Plasma membrane	Mm00435532_m1	65
Gitr	Co-stimulatory survival & function	Plasma membrane / TNFRSF	Mm00437136_m1	89
S1p1	Migration in lymphoid tissues post infection	Plasma membrane	Mm00514644_m1	133
Tim3	Exhaustion (with pd1) during chronic viral infection	Plasma membrane	Mm00454540_m1	98
Tlr2	Proliferation, survival, effector	Plasma membrane	Mm00442346_m1	69
Tlr4	Generating memory $CD8_+$ T cells	Plasma membrane	Mm00445273_m1	87
Notch2	Clearance of acute infection with influenza virus	Plasma membrane	Mm00803077_m1	84
Ccl4	Immune modulation	Extracellular space	Mm00443111_m1	70
Cd70	Co stimulator, ligand for CD27	Extracellular space	Mm00441914_m1	66
Cd40l	Inducer of proliferation, activation	Extracellular space / TNFSF	Mm00441911_m1	120
Fasl	Proliferation and homeostasis (cell death inducer)	Extracellular space / TNFSF	Mm00438864_m1	84
Il-2	Activation	Cytokine	Mm99999222_m1	91
Il-4	Th0 cells to Th2 cells	Cytokine	Mm00445259_m1	79
Il-5	Eosiniophil mediated inflammation	Cytokine	Mm00439646_m1	62
Il-9	Regulating inflammatory immunity	Cytokine	Mm00434305_m1	81
Il-10	Anti-inflammatory	Cytokine	Mm00439614_m1	79
Il-13	Th0 cells to Th2 cells	Cytokine	Mm00434204_m1	56
Il-21	Control of persistent viral infections	Cytokine	Mm00517640_m1	67
Il-22 (Iltifb)	Pro-inflammatory	Cytokine	Mm00444241_m1	86
Mmp2	Regulate T-cell activation	Extracellular space	Mm00439498_m1	62
Mmp9	Regulate T-cell activation	Extracellular space	Mm00442991_m1	76
Ifn-γ	Stimulation during acute viral infection	Cytokine	Mm01168134_m1	100
Tgf-β1	Regulation	Cytokine	Mm01178820_m1	59
Tnf	Regulation of antiviral T-cell response	Cytokine	Mm00443260_g1	61
TraiL	Killing of virally infected cells	Extracellular space / TNFSF	Mm00437174_m1	83
